# Virus-Specific Immune Memory at Peripheral Sites of Herpes Simplex Virus Type 2 (HSV-2) Infection in Guinea Pigs

**DOI:** 10.1371/journal.pone.0114652

**Published:** 2014-12-08

**Authors:** Jingya Xia, Ronald L. Veselenak, Summer R. Gorder, Nigel Bourne, Gregg N. Milligan

**Affiliations:** 1 Department of Microbiology and Immunology, University of Texas Medical Branch, Galveston, Texas, United States of America; 2 Department of Pediatrics, University of Texas Medical Branch, Galveston, Texas, United States of America; 3 Sealy Center for Vaccine Development, University of Texas Medical Branch, Galveston, Texas, United States of America; University of Pittsburgh School of Medicine, United States of America

## Abstract

Despite its importance in modulating HSV-2 pathogenesis, the nature of tissue-resident immune memory to HSV-2 is not completely understood. We used genital HSV-2 infection of guinea pigs to assess the type and location of HSV-specific memory cells at peripheral sites of HSV-2 infection. HSV-specific antibody-secreting cells were readily detected in the spleen, bone marrow, vagina/cervix, lumbosacral sensory ganglia, and spinal cord of previously-infected animals. Memory B cells were detected primarily in the spleen and to a lesser extent in bone marrow but not in the genital tract or neural tissues suggesting that the HSV-specific antibody-secreting cells present at peripheral sites of HSV-2 infection represented persisting populations of plasma cells. The antibody produced by these cells isolated from neural tissues of infected animals was functionally relevant and included antibodies specific for HSV-2 glycoproteins and HSV-2 neutralizing antibodies. A vigorous IFN-γ-secreting T cell response developed in the spleen as well as the sites of HSV-2 infection in the genital tract, lumbosacral ganglia and spinal cord following acute HSV-2 infection. Additionally, populations of HSV-specific tissue-resident memory T cells were maintained at these sites and were readily detected up to 150 days post HSV-2 infection. Unlike the persisting plasma cells, HSV-specific memory T cells were also detected in uterine tissue and cervicothoracic region of the spinal cord and at low levels in the cervicothoracic ganglia. Both HSV-specific CD4^+^ and CD8^+^ resident memory cell subsets were maintained long-term in the genital tract and sensory ganglia/spinal cord following HSV-2 infection. Together these data demonstrate the long-term maintenance of both humoral and cellular arms of the adaptive immune response at the sites of HSV-2 latency and virus shedding and highlight the utility of the guinea pig infection model to investigate tissue-resident memory in the setting of HSV-2 latency and spontaneous reactivation.

## Introduction

HSV-2 infection is widespread globally with an estimated 23.6 million new infections occurring each year [1]. Although disease associated with HSV-2 infection is often limited in severity, more serious manifestations may also occur. HSV-2 present in the birth canal of infected mothers may be passed to neonates during vaginal delivery resulting in serious morbidity and mortality [2], [3]. Vigorous cell-mediated responses are normally responsible for diminishing the severity and duration of HSV-2 disease with immune compromised individuals being more likely to experience severe complications resulting from HSV-2 infection [4], [5]. HSV-2 infection also has been shown to increase the risk of HIV infection and increased HIV shedding is often observed during an active HSV-2 infection in co-infected individuals [6], [7].

HSV-2 has co-evolved with humans and is an extremely successful pathogen, capable of residing long-term in its host and of effective transmission to uninfected individuals. Genital HSV-2 infection of the epithelia spreads to sensory neurons and ultimately results in lifelong latent infection of the innervating sensory ganglia and spinal cord [8], [9], [10]. Once thought to reactivate only occasionally from latency, it is now generally held that reactivation events for most individuals are quite frequent [11] and result in virus shedding often in the absence of overt clinical symptoms. Further, clinical evidence suggests that the period of virus shedding following reactivation is most often of relatively short duration [12], [13] due perhaps to the clearance of virus by HSV-specific T cells residing at the site of previously infected skin [14], [15]. Similar populations of tissue-resident HSV-specific CD4^+^ and CD8^+^ T cells have been found in latently infected trigeminal ganglia of humans [16], [17], [18] and in mice following ocular HSV-1 infection [19], [20]. However, the long term presence and immune function of virus-specific T cells in neural tissues following genital HSV-2 infection has received much less study and less information is available. Sacral ganglia-resident memory cell populations are not currently amenable to study in humans. Infection of mice with fully virulent HSV-2 commonly results in encephalitis and death, precluding easy analysis of the magnitude, phenotype and function of virus specific ganglia- and spinal cord-resident memory T cells in this animal model. The guinea pig model of genital HSV-2 infection represents a unique system to address the nature of both genital-resident and neural tissue-resident immune memory. Genital infection of guinea pigs results in a self-limiting vulvovaginitis with neurologic manifestations mirroring those found in human disease. Virus is transported by retrograde transport to cell bodies in the sensory ganglia and autonomic neurons in spinal cords [10]. During this phase of infection, the virus establishes a latent infection and, similar to humans, the animals undergo spontaneous, intermittent reactivation of virus. HSV-2 recurrences may manifest as clinically apparent disease with erythematous and/or vesicular lesions on the perineum or as asymptomatic recurrences characterized by shedding of virus from the genital tract.

Candidate prophylactic vaccines against HSV-2 have been capable of inducing high levels of systemic, HSV-specific immune responses but have failed to prevent HSV-2 infection or HSV disease in clinical trials [21], [22]. A different vaccine approach is needed to impact HSV-2 disease, perhaps involving development of strong immune responses to HSV-2 at the sites of virus infection. Tissue resident memory immune cells are strategically located to protect against re-infection or interfere with HSV-2 shedding following release of virus after reactivation from latency. The potential for protection conferred by these tissue-resident cell populations has profound implications for HSV-2 vaccine strategies, but the nature of tissue-resident memory immune cells in neural tissues following genital HSV-2 infection, the distribution of immune B and T cells within the reproductive tract, the nature of tissue-resident HSV-specific antibody secreting cells (ASCs) and the functional activity of locally-produced antibody is not well understood. We utilized a genital HSV-2 infection of guinea pigs to address these issues and demonstrate the utility of this approach in assessing the nature of tissue-resident immune cell populations in an animal model that most effectively recapitulates human HSV-2 infection.

## Materials and Methods

### Virus

HSV-2 strain MS stocks were prepared on Vero cell monolayers and stored at -80°C as described previously [23]. Both the replication-defective HSV-2 strain, HSV-2 *dl5*-29, deleted of the HSV DNA replication protein genes UL5 and UL29, and the complementary cell line V529 expressing the UL5 and UL29 proteins [24] were a kind gift of Dr. David Knipe (Harvard Medical School, Boston, MA). Virus stocks were prepared by infection of V529 cells with HSV-2 *dl5*-29 at an MOI of 10. Following development of cytopathic effect (typically 24 hours), cells were subjected to three cycles of freeze-thaw, cell debris pelleted by centrifugation, and the supernatant was aliquoted and frozen at -80°C.

### Guinea pigs

Female Hartley guinea pigs were purchased from Charles River (Burlington, MA) and Strain 13 guinea pigs were obtained from Dr. Marisa St. Claire, National Institutes of Allergy and Infectious Disease, NIH. Female guinea pigs weighing 275 to 300 g were infected by intravaginal (ivag) inoculation with 200 µl of a suspension containing 10^6^ PFU of HSV-2 strain MS as described previously [23]. Animals were observed daily and primary disease severity and frequency of spontaneous recurrent disease were scored daily as described previously [25]. All animals survived the HSV-2 infection and all animals exhibiting either primary or recurrent disease symptoms were included in the studies.

### Ethics statement

This study was carried out in strict accordance with the recommendations in the Guide for the Care and Use of Laboratory Animals of the National Institutes of Health. Guinea pigs were maintained under specific pathogen free conditions and were supplied with food and water *ad libitum* at the Association for Assessment and Accreditation of Laboratory Animal Care-approved animal research center of the University of Texas Medical Branch. All animal research was humanely conducted and approved by the Institutional Animal Care and Use Committee of the University of Texas Medical Branch with oversight of staff veterinarians (Protocol number 0204025B).

### Lymphocyte Isolation from genital tracts and neural tissues

Genital tract and neural tissues (spinal cord and sensory ganglia) were collected from guinea pigs and finely minced. Genital tract tissue was successively digested with DNase I and Dispase II (Roche Diagnostics, Mannheim, German) for 45 min followed by Collagenase (Roche Diagnostics), Hyaluronidase (Sigma-Aldrich, Inc. St. Louis, MO) and DNase I digestion for 60 min at 37°C. Neural tissues were digested with Liberase (Roche Diagnostics) and DNase I for 45 min at 37°C. Lymphocytes from genital tract and neural tissues were isolated by centrifugation over OptiPrep Density Gradients and Percoll Gradients, respectively.

### Enzyme-linked immunospot assay (ELISPOT)

Assays to quantify HSV-specific ASCs were performed as described previously [26] on HSV-glycoprotein-coated plates. To quantify HSV-specific T cells, lymphocytes isolated from spleen, genital tract, or neural tissues of uninfected or HSV-2 infected guinea pigs were stimulated by 48 h culture with splenocyte- or mesenteric lymph node cell-antigen presenting cells (stimulator cells) infected with HSV-2 *dl5*-59 and pulsed with UV-killed HSV-2 to ensure that both MHC class I and class II antigen presenting pathways were engaged to stimulate both virus-specific CD8^+^ and CD4^+^ T cells. Lymphocytes were treated with medium as a control. Cultures were incubated in the presence of recombinant human IL-2 (eBioscience, San Diego, CA) on anti-IFN-γ antibody-coated plates. HSV-specific IFN-γ secreting T cells were detected using monoclonal antibody V-E4 as the capture antibody and biotinylated anti-guinea pig IFN-γ antibody N-G3 as the detection antibody [27] (both antibodies a kind gift of Dr. Hubert Schäfer, Robert Koch Institute, Berlin, Germany). Spots were visualized by incubation with streptavidin peroxidase and AEC (3-Amino-9-ethylcabazole) substrate (Sigma-Aldrich, St. Louis, MO). HSV-specific ASCs and IFN-γ secreting cells were quantified using an ImmunoSpot reader and analyzed with ImmunoSpot software (Cellular Technology Ltd, Cleveland, OH).

### Polyclonal stimulation of B cells

Enriched lymphocyte populations from the spleen, bone marrow, lower genital tract (vagina and cervix), and neural tissues (spinal cord, sensory ganglia) were cultured at 4×10^6^ cells/mL in T cell medium alone or with 4.0 µg/mL LPS and 1.0 µg/mL CpG for 72 hrs. Preliminary studies indicated maximum recovery of antibody secreting cells at 72 h of culture. HSV-specific ASCs from stimulation cultures were quantified on HSV-glycoprotein-coated ELISPOT plates.

### Titration of anti-HSV-2 immunoglobulins

HSV-specific IgA, IgG, IgG1 and IgG2 titers from immune serum or ASC culture supernatants were obtained by ELISA as performed previously [28]. Plate-bound immunoglobulins were detected by addition of polyclonal goat anti-guinea pig IgG (#A60-110A) or polyclonal rabbit anti-guinea pig IgA (#A60-105) (Bethyl Laboratories, Inc., Montgomery, TX) followed by polyclonal HRP-rabbit anti-goat IgG (#A50-100P) or polyclonal HRP-goat anti-rabbit IgG (#A120-101P; Bethyl laboratories, Inc., Montgomery, TX), respectively. IgG1 and IgG2 titers were detected by incubation of plate bound HSV-specific antibodies with polyclonal biotinylated goat anti-guinea pig IgG1 (#ABIN457757) or polyclonal biotinylated goat anti-guinea pig IgG2 (#ABIN457760; antibodies-online Inc., Atlanta, GA) followed by streptavidin peroxidase (Sigma-Aldrich, St. Louis, MO). Normalized optical density readings at 490 nm (OD_490_) obtained from serial dilution of serum or culture supernatants were analyzed by non-linear regression. The end point titer was defined as the serum dilution resulting in an OD_490_ value equivalent to three standard deviations above OD_490_ values from naive sera or medium only. For determination of glycoprotein specificity, ELISA plates were coated with HSV-2 recombinant glycoprotein D (rgD2) and recombinant glycoprotein G (rgG2) purchased from Meridian Life Science, Inc., Memphis, TN.

### Neutralization Assay

Neutralizing antibody titers from immune serum or ASC culture supernatants were determined by a modification of the technique described previously [29]. Neutralizing antibody in supernatants was also demonstrated by adding a series of two-fold dilutions of virus to undiluted ASC supernatant containing Low Tox M rabbit C (Accurate Chemical and Scientific, Westbury, NY) at a final dilution of 1/15. Following incubation at 37°C for one hour, HSV-2 in each sample was quantified by plaque assay on Vero cell monolayers. Vero cells (American Type Culture Collection number CCL-81) were obtained originally from the laboratory of Dr. Lawrence Stanberry, Columbia University School of Medicine, New York, NY).

### Enrichment of CD4^+^ and CD8^+^ cells

Single cell suspensions of genital tract lymphocytes and neural tissue lymphocytes were blocked with anti-Fc RII/III mAb (#553142; BD Biosciences, San Jose, CA) and surface stained with monoclonal mouse anti-guinea pig CD8- fluorescein isothiocyanate (FITC; #MCA752F; clone CT6) or monoclonal mouse anti-guinea pig CD4- phycoerythrin (PE; #MCA749PE; clone CT7; AbD Serotec, Oxford, UK), labeled with anti-FITC or anti-PE microbeads, respectively, (Miltenyi Biotec, Bergisch Gladbach, Germany) and applied to a magnetic column (Miltenyi Biotec) according to the manufacturer's protocol. The phenotype of column-bound and column-flow through populations was assessed by flow cytometry. Data were acquired on a BD FACSCanto II (BD Biosciences) at the UTMB Flow cytometry Core Facility and analyzed using FlowJo software (Tree Star, Ashland, OR). Column enrichment of CD4^+^ T cells commonly resulted in greater than 90% CD4^+^ cells (0.64% CD8^+^ cells). Column enrichment of FITC-labeled CD8^+^ T cells was less efficient (typically 12–29% of collected cells were CD8^+^) although less than 0.5% of obtained cells were CD4^+^.

### Statistical analysis

Statistical differences for B lymphocyte assays, T lymphocyte assays, and antibody titers were determined using Student's *t* test. Differences in cell frequency were determined using the Chi square test. Values for *P*<0.05 were considered significant. All calculations were performed using GraphPad Prism software version 5.0 (GraphPad Software, San Diego, CA).

## Results

### Tissue location of HSV-specific ASC

**Figure 1:**
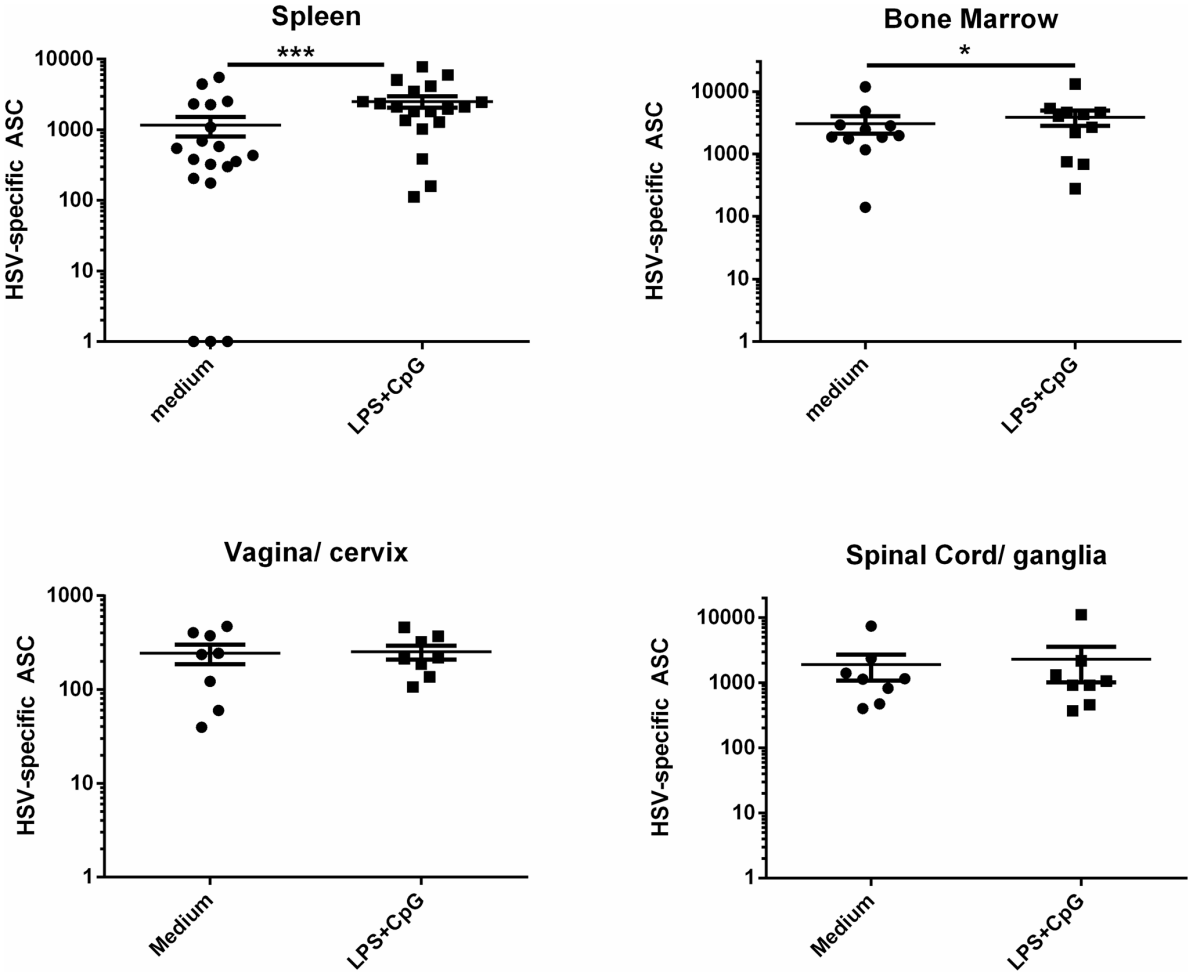
HSV-specific ASC residing long-term in the female genital tract, lumbosacral ganglia and adjacent spinal cord are predominantly plasma cells. Hartley guinea pigs were infected ivag with HSV-2 strain MS. Lymphocytes from the spleen, bone marrow, vagina/cervix, and lumbosacral ganglia and adjacent spinal cord of individual animals were harvested between days 49–70 after infection and stimulated with medium or LPS/CpG and HSV-specific ASC quantified by ELISPOT. Each data point represents results from an individual animal. Results shown include tissues from three experiments for spleen and bone marrow and two experiments for vagina/cervix and spinal cord/ganglia. HSV-specific ASCs from tissues of uninfected animals were always less than five ASC/tissue. (* *P*<0.05; *** *P*<0.0001, Student's *t* test).

We previously demonstrated that HSV-specific ASC were detected long-term at the site of HSV-2 latency in the sensory ganglia and at the site of virus shedding in the female genital tract in guinea pigs infected ivag with HSV-2 [28]. We hypothesized that these ASC represented long-lived plasma cells existing in survival niches at these sites of chronic inflammation although an alternative explanation is that these ASC derived from tissue-resident memory B cells as a result of periodic exposure to HSV antigens released during reactivation events. HSV-specific memory B cells were detected functionally because antibodies for guinea pig memory B cell markers are not currently available. Memory B cells, but not plasma cells, become activated and differentiate into ASC upon stimulation with polyclonal activating agents such as TLR-agonists [30], [31]. The presence of memory B cells in the culture is therefore detected as a TLR-agonist-induced increase in the number of antigen-specific, ASCs. To test for the presence of memory B cells at peripheral tissues, we stimulated lymphocyte populations isolated from bone marrow, spleen, vagina/cervix, or spinal cord/sensory ganglia of previously infected guinea pigs with a combination of LPS and CpG oligonucleotides and quantified HSV-specific ASC by ELISPOT. Hartley guinea pigs were infected with HSV-2 strain MS. Fourteen of the 19 animals used for assessment of HSV-specific ASC were scored for both primary and recurrent HSV-2 disease to document HSV disease. Primary HSV-2 disease was observed in 12 of 14 animals with a mean disease score of 5.4±1.1 and at least one recurrent lesion was detected in 12 of 14 infected animals between days 15 PI to the day of euthanasia resulting in a mean recurrent disease score of 1.2±0.26. Tissues were harvested from individual animals between days 49–70 after infection. Less than five HSV-specific ASCs were detected in all tissues from uninfected control animals. However, as shown in **Fig. 1**, HSV-specific ASCs were detected in all tissues from HSV-2 infected animals following control treatment. Significantly greater numbers of HSV-specific ASC were detected in LPS/CpG-stimulated splenocyte cultures compared to medium-stimulated cultures (*P*<0.0001, Student's *t* test) indicating the presence of HSV-specific memory B cells. Additionally, a smaller but still significant increase in ASC number was also detected in LPS/CpG-stimulated bone marrow cells compared to stimulation with medium alone (*P*<0.05, Student's *t* test). In contrast, there was no difference in HSV-specific ASC number following medium- or LPS/CpG-stimulation of lymphocytes isolated from the vagina/cervix or spinal cord/sensory ganglia. Therefore, HSV-specific memory B cells were readily detectable in the spleen and to a lesser extent in the bone marrow of HSV-2 infected guinea pigs but not at the peripheral sites of HSV-2 infection. These results suggest that the vast majority of HSV-specific ASC present in the lower genital tract, lumbosacral ganglia and adjacent spinal cord on days 49-70 post infection represented a persisting population of plasma cells.

**Figure 2:**
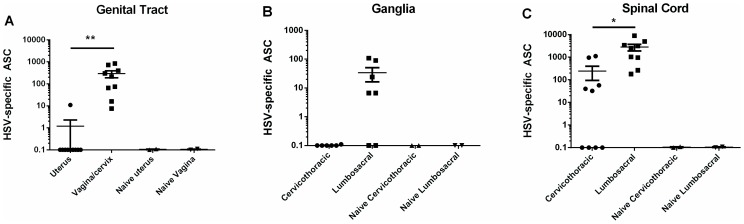
Location of HSV-specific, tissue-resident ASCs in guinea pigs infected previously with HSV-2. Hartley guinea pigs were infected ivag with HSV-2 strain MS and lymphocytes isolated from the indicated tissues on days 48–57 post infection. HSV-specific ASCs were quantified by ELISPOT on HSV-2 glycoprotein-coated plates. Each data point represents results from an individual animal. (* *P*<0.05; ** *P*<0.01, Student's *t* test).

To more precisely define the tissue distribution of HSV-specific ASC, lymphocytes from uterus, vagina/cervix, cervicothoracic sensory ganglia, cervicothoracic spinal cord, lumbosacral sensory ganglia and the adjacent spinal cord were isolated from individual animals between 49–70 days post infection and HSV-specific ASC quantified by ELISPOT. As shown in **Fig. 2**, HSV-specific ASCs were detected in lymphocyte populations isolated from tissues of infected- but not uninfected-guinea pigs. The presence of genital tract-resident, HSV-specific ASC was limited mainly to the vagina and cervix as only a very low number of HSV-specific ASCs were isolated from the uterus of a single infected animal (**Fig. 2A**). HSV-2 genomes were detected at this time period in the vagina/cervix tissue from two of four infected animals which most likely represented HSV-2 shedding; however, no virus genomes were detected in the uterus. Similarly, HSV-specific ASCs were detected in the lumbosacral ganglia innervating the genital tract of five of seven animals, but not in the cervicothoracic ganglia of infected guinea pigs (**Fig. 2B**). HSV-specific ASCs were detected in spinal cord tissue isolated from the cervicothoracic region in five of ten infected animals whereas significantly higher numbers of HSV-specific ASC were detected in the lumbosacral region of the spinal cord in nine of ten infected animals (*P*<0.05, Student's *t* test, **Fig. 2C**).

### Characteristics of HSV-specific antibody secreted by ASC isolated from lymphoid and peripheral tissues of HSV-2 infected guinea pigs

**Figure 3:**
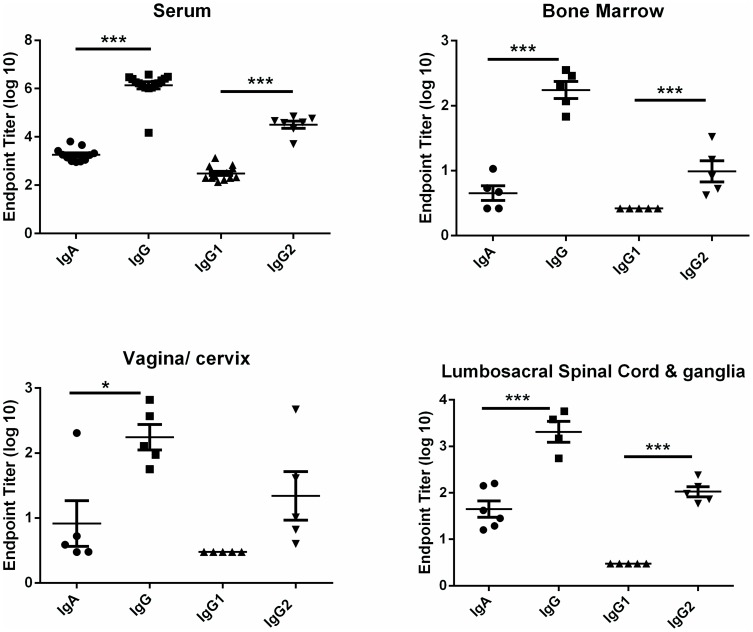
Isotype and IgG subclass of HSV-specific antibodies produced by ASCs isolated from lymphoid tissue and non-lymphoid sites of HSV-2 infection. Hartley guinea pigs were infected ivag with HSV-2 strain MS, serum was collected and lymphocytes were isolated from the indicated tissues on days 48–57 post infection. Supernatants from ASC cultures were collected on day three after ASC isolation. The immunoglobulin isotype and IgG subclass of HSV-specific antibodies from serum and ASC supernatant were determined by ELISA. Each data point represents ASC supernatant from an individual animal. (* *P*<0.05; ***P<0.0001, Student's *t* test).

**Figure 4:**
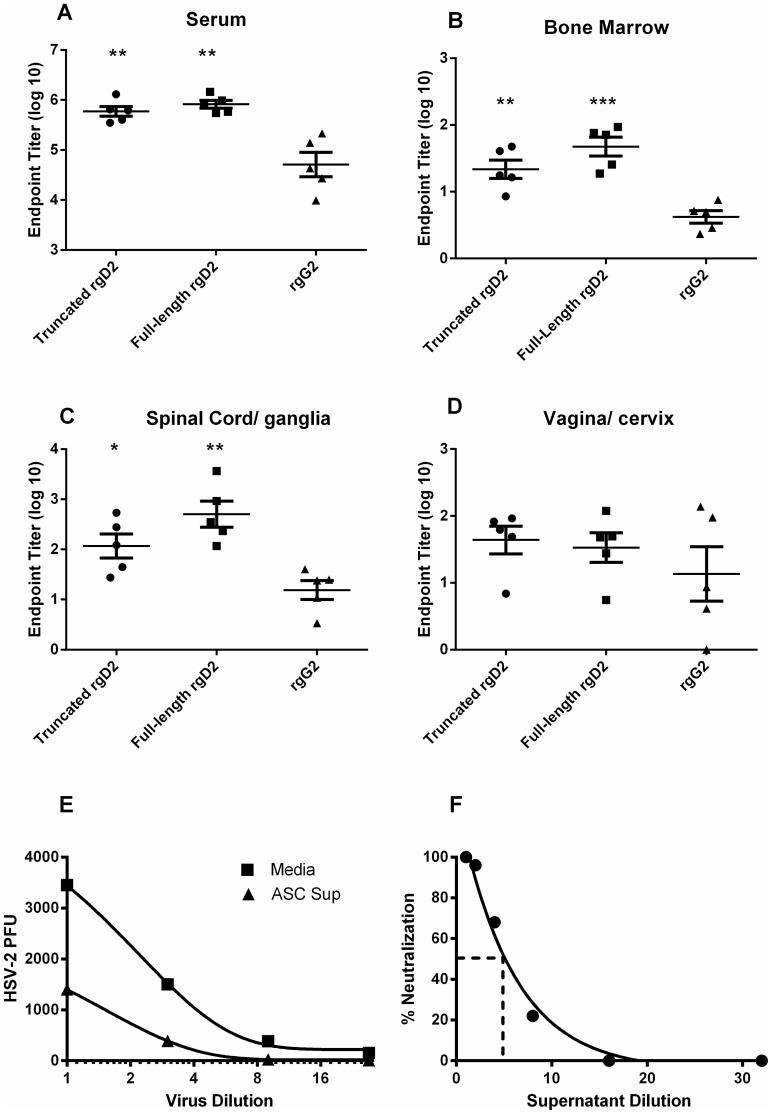
IgG antibody from tissue-resident ASC isolated from HSV-2-infected guinea pigs is reactive with HSV-2 glycoproteins and neutralizes HSV-2. Hartley guinea pigs were infected ivag with HSV-2 strain MS, serum was collected and lymphocytes were isolated from the indicated tissues between days 48–57 post infection. Supernatants from ASC cultures were collected on day three after isolation and the endpoint titer of antibodies reactive with full-length recombinant HSV-2 gD (rgD2), truncated rgD2, or truncated recombinant HSV-2 rgG (rgG2) was determined by ELISA. (*P<0.05; ** *P*<0.01; *** *P*<0.001 compared to HSV-2 gG2 titer). Virus neutralization was detected by incubating a constant virus titer in serial dilutions of ASC supernatant to determine a 50% neutralizing titer (E) or by incubating serial dilutions of HSV-2 virions in undiluted ASC supernatant (F). Results shown are from a representative experiment of three performed.

To characterize the antibody produced by tissue-resident ASC and to determine if the isotype and IgG subclass profile of antibody from HSV-specific ASC isolated from peripheral sites of HSV-2 infection were similar to HSV-2-specific antibodies in immune serum, ASC were isolated from selected tissues and the antibody-containing culture supernatant was harvested after three days of culture for analysis by ELISA (**Fig. 3**). In sera from immune animals, titers of HSV-specific IgG were significantly higher than IgA titers (*P*<0.001, Student's *t* test) and the IgG2 subclass predominated the response. A similar isotype profile was apparent for HSV-specific antibody produced by bone marrow-, vagina/cervix-, and spinal cord/ganglia ASC. Antibody was also tested for binding to recombinant HSV-2 glycoproteins representing known targets of antibodies in convalescent sera. As shown in **Fig. 4**, HSV-specific serum IgG bound at a nearly equivalent level to full-length or truncated recombinant HSV-2 glycoprotein D (rgD2) while binding to recombinant HSV-2 glycoprotein G (rgG2) was detected at a significantly lower level. The same binding pattern was observed for IgG produced by bone marrow- and spinal cord/ganglia- ASC (**Fig. 4B, C**). The level of IgG produced by ASC from the vagina/cervix was more variable and therefore binding to the two forms of rgD2 and rgG2 was not significantly different (**Fig. 4B**). HSV-2 neutralizing antibody was present at a titer of 1∶1023±196 in the serum of HSV-2 infected guinea pigs. Functional HSV-2-neutralizing antibody was also produced by ASCs cultured from the lumbosacral ganglia and spinal cords of HSV-2 infected animals (**Fig. 4E, F**). Incubation of HSV-2 with supernatants of spinal cord/ganglia-resident ASC decreased viral titers over a range virus inoculums compared to the medium control (**Fig. 4E**). Additionally, ASC supernatant could be diluted approximately 5-fold and retain the ability to neutralize 50% of input virus (**Fig. 4F**). Neutralizing antibody was not detected in supernatants of ASC from genital tracts perhaps due to an approximately 10-fold lower number of HSV-specific ASC isolated from genital tract ASC compared to lumbosacral spinal cord ASC (**Fig. 2A** compared to **Fig. 2C**). To our knowledge, these studies represent the first characterization of HSV-specific antibodies produced by neural tissue-resident ASC during HSV latency.

### HSV-specific, tissue-resident memory T cells present at the sites of HSV-2 latency and virus shedding

**Figure 5:**
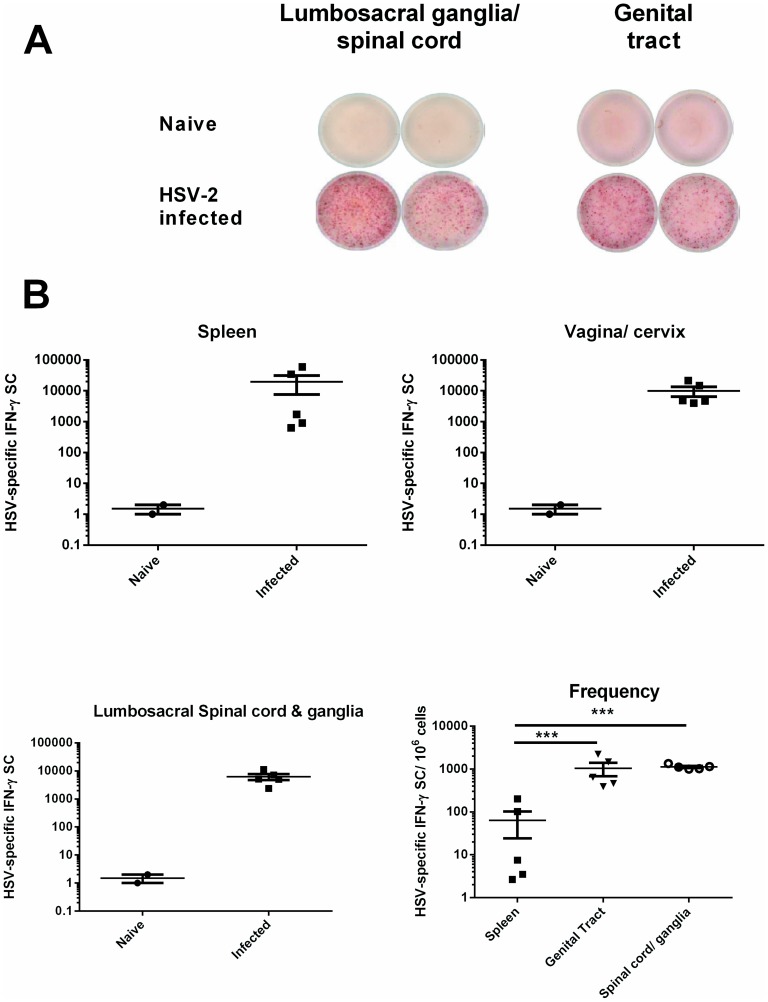
Detection and quantification of HSV-specific IFN-γ SCs in spleen, genital tract and neural tissues of HSV-2 infected guinea pigs. Lymphocytes from the indicated tissues of HSV-2-infected Strain 13 guinea pigs (n = 5) were harvested on day 7 post infection. A) IFN-γ ELISPOT of lymphocytes harvested from sensory ganglia/spinal cord and genital tract. B) Total number and frequency of HSV-specific IFN-γ SC in the spleen, genital tract, sensory ganglia/spinal cord of HSV-2 infected guinea pigs. Each data point represents results from an individual animal. *** *P*<0.0001, Chi square.

The activation and infiltration of HSV-specific T cells producing IFN-γ at the peripheral sites of acute HSV-2 infection in the genital tract and sensory ganglia has been observed in murine models of genital HSV-2 infection [29], [32], [33] and from HSV lesion-derived human T cells [34], [35]. To further characterize T cell immunity in the genital tract, lumbosacral ganglia and spinal cord, Strain 13 guinea pigs were inoculated ivag with HSV-2 strain MS. All animals experienced primary HSV disease resulting in a mean disease score of 5.7±1.1. On day 7 post infection, HSV-specific, IFN-γ-secreting T cells from the spleen, vagina/cervix, and lumbosacral ganglia and the adjacent spinal cord were quantified using a pair of previously described anti-guinea pig IFN-γ -specific monoclonal antibodies [27] in an IFN-γ ELISPOT assay. Lymphocytes from the indicated tissues were stimulated by culture with stimulator cells infected with HSV-2 *dl5*-29 and pulsed with UV-inactivated HSV-2 to ensure that both MHC class I and class II antigen presenting pathways were engaged to stimulate both CD4^+^ and CD8^+^ T cells. As shown in **Fig. 5A-B**, IFN-γ secreting cells (SC) were readily detected in tissues from HSV-infected, but not uninfected guinea pigs. HSV-specific IFN-γ SCs detected in the spleen, vagina/cervix, and spinal cord/lumbosacral ganglia of HSV-infected guinea pigs were over four logs greater than in uninfected animals (**Fig. 5B**). Although the total number of HSV-specific, IFN-γ SCs was similar in all three tissues, the frequency of these cells isolated from the spleen was somewhat variable among individual animals and significantly lower overall than the frequencies of HSV-specific, IFN-γ SC isolated from the vagina/cervix or spinal cord/ganglia (*P*<0.0001, Chi square).

**Figure 6:**
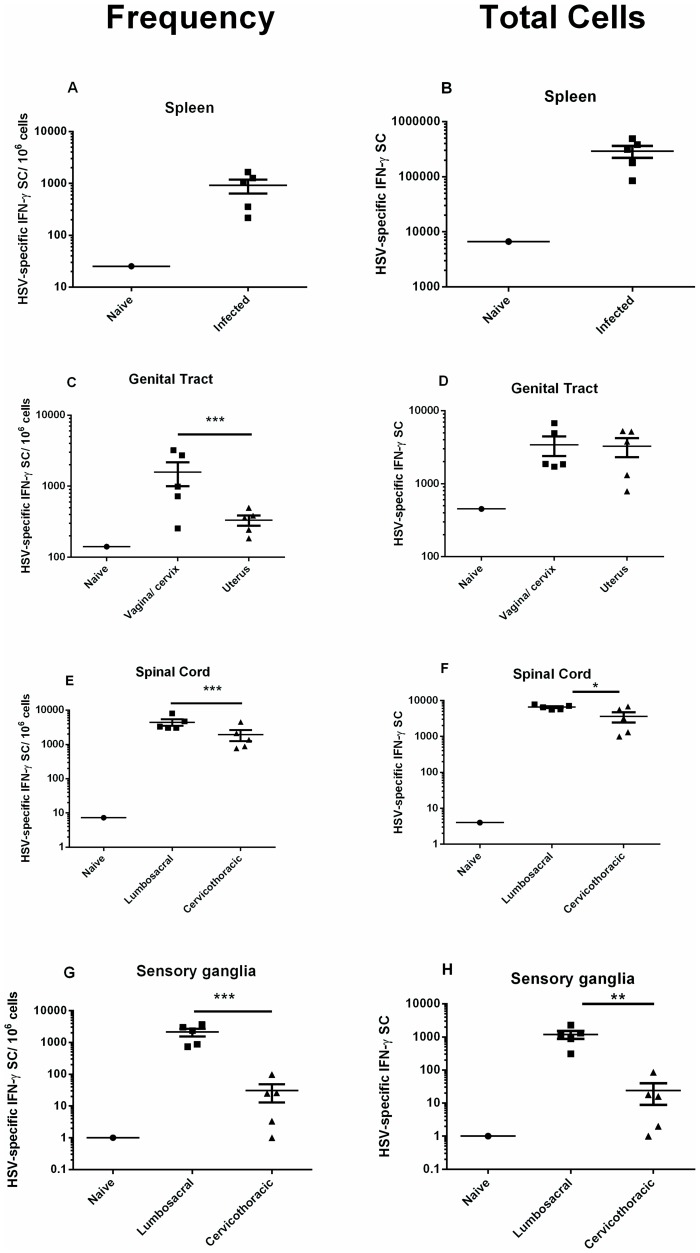
Tissue distribution of HSV-2 specific memory T cells following genital HSV-2 infection of guinea pigs. Hartley guinea pigs were infected ivag with HSV-2 strain MS and lymphocytes isolated from the indicated tissues on days 99-150 post infection. HSV-specific, IFN-γ SCs were detected and quantified by IFN-γ ELISPOT. The frequency of HSV-specific, IFN-γ SC per 10^6^ cells is given in **A,C,E,G** and the total number of HSV-specific, IFN-γ SC is given in **B,D,F,H** for each tissue. Each data point represents results from an individual animal. (* *P*<0.05; ** *P*<0.01, Student's *t* test; *** *P*<0.0001, Chi square).

The IFN-γ ELISPOT assay was modified for use in outbred animals and used to detect and quantify HSV-specific memory T cells from tissues of HSV-2-infected Hartley guinea pigs. Animals were inoculated ivag with HSV-2 strain MS and the primary and recurrent disease were scored. Ten of the 11 animals utilized for memory T cell analysis experienced primary HSV disease (Mean score 6.7±1.0) and 10 of 11 animals experienced at least one recurrent lesion (Mean score 5.2±0.7) between day 15 and the time of euthanasia (day 99–150). Tissues were harvested from individual animals between days 99–150 post infection. Cells from spleen, vagina/cervix, uterus, lumbosacral ganglia, cervicothoracic ganglia and the corresponding adjacent region of spinal cord were stimulated with stimulator cells infected with HSV-2 *dl5*-29 and pulsed with UV-inactivated HSV-2 to stimulate both CD4^+^ and CD8^+^ memory T cells. As shown in **Fig. 6A-B**, HSV-specific memory T cells were readily detected in the spleen of HSV-2 infected animals (mean 291,426±72,324 IFN-γ SC/spleen). HSV-specific memory T cells were also detected in both the upper and lower genital tract of all animals tested (**Fig. 6C, D**). Although present at significantly higher frequency in the vagina/cervix than in the uterus (P<0.0001, Chi square; **Fig. 6C**), overall, the total number of HSV-specific memory T cells was not different between these tissues (**Fig. 6D**) reflecting differences in the yield of lymphocytes isolated from these tissues. HSV-specific memory T cells were detected over the entire length of the spinal cord. However, the frequencies of tissue-resident, memory T cells detected in lumbosacral region spinal cord and cervicothoracic region spinal cord were significantly different (*P*<0.0001, Chi square; **Fig. 6E**) and the total number of HSV-specific memory T cells was significantly greater in spinal cord tissue from the lumbosacral region (*P*<0.05, Student's *t* test). Similarly, HSV-specific memory T cells were detected in both the lumbosacral and cervicothoracic ganglia. Importantly, virus-specific memory T cells were also detected at significantly higher frequency in the lumbosacral compared to cervicothoracic ganglia (*P*<0.0001, Chi square; **Fig. 6G**) and the total number of HSV-specific memory T cells was significantly greater in lumbosacral ganglia compared to cervicothoracic ganglia (*P*<0.01, Student's *t* test, **Fig. 6H**).

**Figure 7:**
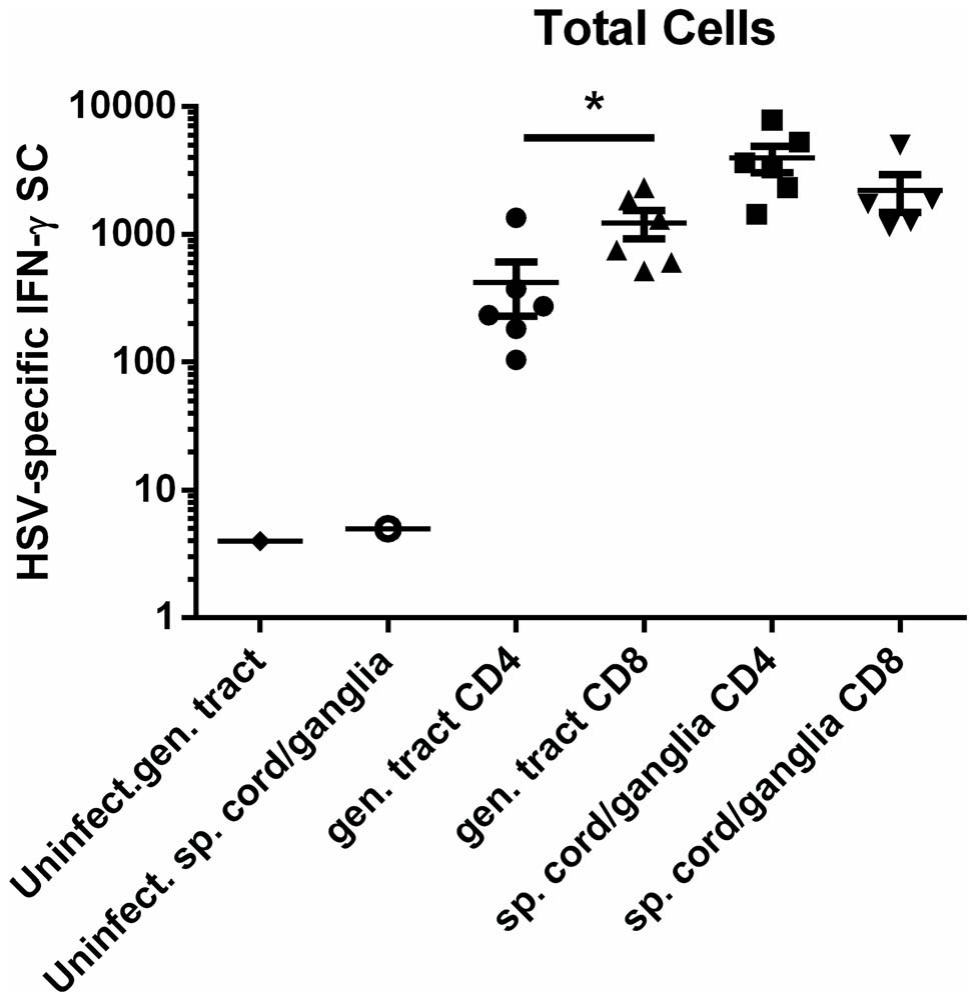
Presence of CD4^+^ and CD8^+^ HSV-specific memory T cells in genital tracts and neural tissue of HSV-2 infected guinea pigs. Hartley guinea pigs were infected ivag with HSV-2 strain MS and lymphocytes isolated from the indicated tissues on days 99–150 post infection. Enriched populations of CD4^+^ and CD8^+^ T cells were obtained using a magnetic-based kit. HSV-specific, IFN-γ SCs were detected and quantified by IFN-γ ELISPOT. Each data point represents results from an individual animal. (* *P*<0.05 Student's *t* test).

Lymphocytes were harvested from peripheral tissues of individual infected animals between days 99-150 post infection and passed over selection columns to enrich for CD4^+^ or CD8^+^ subsets to further characterize the T cell memory response. CD4^+^ enriched populations were stimulated with stimulator cells pulsed with UV-inactivated HSV-2 while CD8^+^ enriched populations were stimulated with stimulator cells infected with HSV-2 *dl5*-29. HSV-specific memory T cells representing both CD4^+^ and CD8^+^ subsets were readily detected in the vagina/cervix of HSV-2-infected guinea pigs at 2-6 months post infection (**Fig. 7**). HSV-specific CD4^+^ T cells were detected in the vagina/cervix at significantly lower total cell number compared to HSV-specific CD8^+^ T cells (*P*<0.05, Student's *t* test). Both CD4^+^ and CD8^+^ HSV-specific memory T cells were maintained in the sensory ganglia/spinal cords of HSV-2 infected guinea pigs (**Fig. 7**). The total number of ganglia/spinal cord-resident, HSV-specific CD4^+^ and CD8^+^ T cells was not different demonstrating the maintenance of both subsets of virus specific memory T cells at the sites of HSV-2 latency during the initial months after HSV-2 infection.

## Discussion

HSV-specific, tissue-resident CD4^+^ and CD8^+^ memory T cells have been detected in genital skin at the site of previous HSV-2 infection [15], [36], [37] and at the site of HSV latency in the trigeminal ganglia of HSV-1-infected humans and mice [16]-[20]. Using a guinea pig model of HSV-2 genital infection, we extend these results and show here that a full complement of adaptive immune cells including HSV-specific B cells, CD4^+^ T cells, and CD8^+^ T cells resides in the genital tract site of HSV-2 infection and at the site of latency in the sensory ganglia. Additionally, consistent with the recent observation that genital HSV-2 infection results in virus infection of autonomic neurons in the spinal cord [10], we show here that the development and maintenance of all these HSV-specific memory immune cells extends also to HSV-2 infected spinal cords. These tissue-resident populations are appropriately positioned to play a rapid and critical role in modulation of HSV-2 reactivation and to control HSV-2 shedding. The induction of these tissue-resident memory cells by immunization would most likely be an important contributor to successful immunization strategies against HSV-2.

The cellular components comprising B cell memory are quiescent memory B cells and antibody-secreting, long lived plasma cells (LLPC). Memory B cells normally reside in secondary lymphoid tissue although they have also been detected at peripheral sites of previous virus infection [38]. Because immune reagents that distinguish memory B cells are not yet available for guinea pigs, we utilized the ability of memory B cells to become activated and secrete antibody in response to polyclonal stimulation to phenotypically identify these cells. TLR agonist-stimulated memory B cells were readily detected in the spleen and to a lesser extent in the bone marrow of infected animals but not in lymphocytes isolated from genital or neural tissues. While the assay may have lacked the sensitivity to absolutely exclude the presence of memory B cells in these tissues, the results strongly suggest that the majority of the HSV-specific ASC detected in these tissues represent persistent plasma cell populations. LLPC reside primarily in lymphoid tissues such as the spleen or bone marrow and are responsible for maintaining serum antibody titers. However, populations of LLPCs have also been detected at sites of chronic inflammation including the nervous systems of humans infected with polio [39] or animals persistently infected with coronaviruses [40], [41]. HSV-specific ASCs were predominantly detected at peripheral tissue sites that had experienced active HSV-2 infection suggesting that antigen and/or active inflammation are required for the establishment of these populations. The influence of intermittent exposure to HSV antigen following spontaneous HSV-2 reactivation on the population dynamics of these ASC is not yet known. However, this population is most likely maintained in an antigen-independent fashion in survival niches at sites of chronic HSV infection in which survival stimuli such as IL-6 or CXCL12 are consistently present [42].

Both IgG and IgA have been detected in serum and vaginal secretions of guinea pigs following ivag infection with HSV-2 [43] or Chlamydia [44]. Although HSV-specific IgA was consistently detected, IgG antibody represented the predominant isotype produced by genital tract resident ASC as reported in both mice and humans [45], [46]. Additionally, the predominance of IgG2 expression by antibodies derived from peripheral tissue ASC mirrored that of serum antibody and bone marrow-derived ASC. Antibody produced by genital- and neural tissue-resident ASC recognized rgD2 and rgG2 and HSV-specific antibody produced by neural tissue-resident ASC was capable of neutralizing HSV-2. Together these results demonstrate the ability of these cells to produce functionally relevant antibody at sites of chronic HSV infection.

IgG may enter vaginal secretions by transudation from the serum or by receptor-mediated transport across the vaginal epithelium by FcRN [47] and does not require the presence of genital-resident ASC. However, IgG production by genital-resident, virus-specific ASC would increase the local concentration of virus-specific IgG resulting in increased movement of this antibody into vaginal secretions via simple diffusion or FcRN-mediated transport. The role for local antibody in protection of humans against HSV-2 infection or HSV disease is uncertain. The presence of HSV-specific maternal antibody in vaginal secretions decreases the risk of HSV-2 acquisition by neonates [2]. By analogy, locally-produced HSV-specific antibodies in mucosal secretions might prevent or limit virus infection. It has been shown that re-infection of ganglia is extremely difficult to achieve in previously infected animals due in part to the presence of pre-existing HSV-specific antibody [48], [49]. Locally-produced antibody in neural tissues may limit virus access to neurons, limit spread and acute replication of virus within the ganglia, and, upon reactivation from latency, interfere with virus spread from infected nerve ending to epithelial cells [50]–[52]. Plasma cells are also a source of cytokine production in inflamed tissues and B cell-derived cytokines such as IL-6, IL-12, TGF-β, and IL-10 that may play a role in regulating the inflammatory response in sites chronically infected with HSV-2. Given these functions of HSV-specific B cells, the populations of HSV-specific ASC we detected long term in genital tract, latently-infected sensory ganglia and spinal cords seem strategically located to play an active role in modulation of recurrent disease and shedding or in protection of genital and neural tissue against re-infection upon subsequent HSV-2 exposure.

In humans, HSV-specific CD8^+^ T lymphocytes have been isolated from HSV lesion material and from genital skin at the dermal-epidermal junction after HSV-2 infection [14], [15], [36]. HSV-specific CD4^+^ T cells have also been detected in the vaginal epithelium long-term after HSV-2 genital infection of mice [29], [32], [53] and have also been isolated from HSV lesion material from humans [34], [35]. The development of genital tract-resident memory T cells in guinea pigs as a result of genital HSV-2 infection is apparently very similar and it is of note that a strong IFN-γ response is detected upon stimulation of these cells as has been detected in both mice and humans [29], [36], [37]. Prophylactic vaccination to induce genital tract-resident memory T cells would theoretically provide protection against the initial infection whereas therapeutic vaccination to enhance a genital tract-resident memory T cell population might aid in modulating virus shedding from the genital tract of those individuals already infected.

HSV-specific, IFN-γ secreting effector T cells were detected in the lumbosacral ganglia and spinal cord during acute HSV-2 infection. Importantly, large populations of HSV-specific, CD4^+^ and CD8^+^ memory T cells were detected at these sites up to 150 days after HSV-2 infection. The maintenance of memory T cell populations at the sites of HSV-2 latency following genital HSV-2 infection has not been extensively studied previously. Sacral ganglia-resident T cell populations are not readily accessible in humans and infection of mice with HSV-2 frequently results in mortality complicating long-term study of memory T cells. We know from ocular HSV-1 infection of mice that HSV-specific CD8^+^ T cells surround HSV-1 infected neurons in the trigeminal ganglia of mice [20], [54] and may be involved in the maintenance of HSV latency via non-lytic mechanisms involving IFN-γ and release of granzyme B [54], [55]. Similarly, HSV-2 –specific CD4^+^ T cells have been shown to play a role in resolution of an acute virus infection of the lumbosacral ganglia neurons following genital HSV-1 infection [33]. The role of these neural tissue-resident memory T cells in modulating HSV-2 reactivation and how the population dynamics of these populations is influenced by chronic exposure to HSV-2 is currently unclear. Utilization of this animal model in which spontaneous reactivation of HSV-2 occurs should help resolve these issues.

In contrast to the tissue location of HSV-specific ASC, the HSV-specific memory T cells were more broadly dispersed in both the genital tract and neural tissues. These results suggest differences in either the inflammatory milieu or antigen load between the T and B cell experiments or different sensitivities of our assays to detect HSV-specific memory T and ASC populations. Our results also demonstrate that beyond the ganglia site of HSV-2 latency, HSV-specific resident memory T cells become established in the spinal cord following HSV-2 genital infection. Ohashi et al. [10] detected the greatest quantities of latent HSV-2 genomes in the sacral region of the spinal cord. Consistent with this finding, although HSV-specific memory T cells were detected throughout the entire spinal column, the highest frequency and the greatest total number of HSV-specific memory T cells were detected in the lumbosacral region. The current study therefore extends previous results regarding the nature of immune memory in the sacral ganglia and spinal cord sites of latency following genital HSV-2 infection.

Guinea pigs infected ivag with HSV-2 experience intermittent, spontaneous reactivation events resulting in recurrent virus shedding and development of clinical symptoms remarkably similar to humans. A roadblock to studying immune control of HSV-2 in this model has been the lack of reagents for immune response proteins. We previously isolated genital tract resident lymphocytes to detect HSV-specific ASC in the genital tracts and neural tissue of HSV-2-infected guinea pigs for time periods up to 8 months after genital infection [28]. Our current studies extend these previous results and demonstrate that ASC releasing functionally protective, HSV-specific antibody are present at the sites of HSV-2 latency and sites of virus shedding. The nature of immune memory at sites of HSV-2 infection and latency was further clarified by development of an IFN-γ ELISPOT to detect and quantify HSV-specific effector and memory T cells and demonstrate vigorous HSV-specific memory T cell responses at these epithelial and neural sites. Future studies utilizing the guinea pig HSV-2 infection model will allow characterization of important properties of these cells and test immunization regimens to induce genital tract resident T cell populations in the context of virus reactivation and shedding that most effectively models human HSV-2 infection.
